# Patterns and Outcomes of Craniomaxillofacial Trauma in Puerto Rico: Insights From a Five-Year Retrospective Study

**DOI:** 10.7759/cureus.82115

**Published:** 2025-04-11

**Authors:** Raúl Y Ramos-Sánchez, Hermes A Aponte Rivera, Claudia B Sotomayor Rivera, Arnaldo Y Figueroa Tejada, Daniela Bresciani-Padilla, David A Febre-Alemañy, Francisco Del Valle-Díaz, José López-Fontanet, Julián Zayas-Vélez, Francisco Biaggi-Huyke, Gabriela Pomales-Díaz, Guillermo López Torres, Jan Ruiz-Núñez, Carlos Viera-Maldonado, Miciely Aponte-Reyes, Elvis Santiago-Rodríguez, Ángel Rivera-Barrios

**Affiliations:** 1 Otolaryngology - Head and Neck Surgery, University of Puerto Rico, Medical Sciences Campus, San Juan, PRI; 2 General Surgery, University of Puerto Rico, Medical Sciences Campus, San Juan, PRI; 3 Radiology, University of Puerto Rico, Medical Sciences Campus, San Juan, PRI; 4 Surgery, San Juan Bautista School of Medicine, Caguas, PRI; 5 Ophthalmology, University of Puerto Rico, Medical Sciences Campus, San Juan, PRI; 6 General Surgery, Ponce Health Sciences University, Ponce, PRI; 7 Internal Medicine, HCA Florida Oak Hill Hospital, Tampa, USA; 8 Plastic Surgery, University of Puerto Rico, Medical Sciences Campus, San Juan, PRI

**Keywords:** cranial fracture, craniomaxillofacial trauma, facial fracture, hispanic population, injury epidemiology

## Abstract

Background

Craniomaxillofacial (CMF) trauma is a significant problem in the United States, with estimated costs of nearly one billion dollars annually. Facial fractures occur based on factors such as facial structure, the direction and intensity of the impact, and the mechanism of injury. The most frequent facial fractures include the nose, orbits, zygomatic complex, mandible, maxilla, and frontal bone. Additionally, demographic, social, cultural, and environmental factors can contribute to particular trauma mechanisms like falls and motor vehicle accidents (MVAs), leading to different CMF injury rates among populations. Overall, CMF traumas have a significant potential for morbidity and mortality. This study aims to provide the first overview of the prevalence of CMF trauma in Puerto Rico.

Methods

This retrospective study includes patients aged 0-100 who presented with CMF trauma from 2018 to 2022 to the only trauma center in Puerto Rico. Demographic and clinical data were collected, including the mechanism of injury, craniofacial structures involved, treatment, and outcomes. Frequencies of demographic and clinical data were documented, and statistical analysis using one-way ANOVA and t-tests was performed.

Results

A total of 1,102 patients (83.1% male and 16.4% female) with CMF injuries were included. The mean age of the group was 40.67 years. The most common mechanisms were non-car-related MVA (23.6%), car-related MVA (22.9%), pedestrian accidents (18.4%), falls (15.9%), and gunshot wounds (10.4%). Cranial fractures occurred in 32.7% of patients with the following affected regions: temporal (16.2%), frontal (10.6%), parietal (7.8%), and occipital (5.3%). Facial fractures occurred in 70% of patients with the following affected regions: middle face including maxilla, nose, zygoma, and orbits (61.8%), lower face including mandible (17.7%), and upper face including frontal bone (9%). Approximately 19.8% of patients with CMF fractures underwent surgical management. The mortality rate in the cohort was 11.8%. The Glasgow Coma Scale (GCS) and Injury Severity Score (ISS) were significantly worse in patients with cranial (p < 0.001) and/or facial (p < 0.001) fractures when compared to patients who suffered from CMF traumas without fractures.

Conclusion

To our knowledge, this is the first study characterizing CMF traumas in Puerto Rico. The majority of the affected patients were male and belonged to the adult population. Common etiologies of injury were comparable to others reported in the literature, including MVA, falls, and gunshot wounds. Facial fractures were more prevalent than cranial fractures in our cohort. Patients with high-severity injuries were more likely to be managed surgically. By establishing the epidemiological picture of CMF traumas in Puerto Rico, public health and clinical efforts may be employed to allow for improved patient outcomes.

## Introduction

Craniomaxillofacial (CMF) trauma is described as any injury involving the soft tissues of the face, neck, and scalp, as well as the bony tissue of the facial skeleton, including the facial bones or the cranium [1]. The severity varies depending on the etiology of the trauma and may occur in isolation or in combination with injuries to other parts of the body. The treatment of these patients will vary depending on their location and severity, ranging from more conservative interventions such as external fracture reductions to other more invasive surgical approaches.

CMF injuries are one of the most common forms of trauma. Vehicular trauma is one of the leading etiologies of facial injury, accounting for 11%-85% of cases [2]. In addition, five leading causes of injury exist, such as work, traffic, assaults, sports, and activities of daily living (ADL), which account for up to 25%-30% of craniofacial trauma cases. According to a study by Romeo et al. [3], women are more likely to undergo facial trauma from both physical and sexual assaults. On the other hand, men are more likely to experience trauma from motor vehicle accidents (MVAs) and falls. This difference in etiologies between men and women can also account for the differences in the management, mortality, complications, and severity of injuries.

Facial fractures occur based on various factors such as the patient's facial morphology, the vector in which force was applied to the facial bones, and the bone age of the patient. Additionally, socioeconomic and environmental factors can contribute to increased risk for falls, assaults, and MVAs, leading to different rates of CMF injuries among different patient populations. For example, one study by Wright et al. showed that MVAs were significantly more common in low-income and high-poverty areas [4]. Additionally, assaults and road traffic accidents have been classified as the predominant causes of facial fractures in disadvantaged patient populations [5]. The former may be partially explained by the fact that, for example, intimate partner violence, notably among women, is more prevalent in low socioeconomic status groups [6]. Moreover, socioeconomic deprivation has been identified as a strong predictor of facial fractures requiring surgery for both men and women [5].

Overall, CMF trauma has a significant potential for high morbidity and mortality. Additionally, these are known to cause substantial distress in those who survive them due to the long-term consequences, both functionally and esthetically [3]. Understanding the patterns of craniofacial injuries assists healthcare providers in planning and managing the treatment of traumatic craniofacial injuries [1]. These statements highlight the importance of identifying the prevalence of CMF fractures in the Puerto Rican population since our findings may help identify those at risk and contribute to developing strategies to minimize the impact of these types of fractures on our community.

As of now, to our knowledge, there are no studies regarding CMF trauma and fractures in Puerto Rico. Therefore, we sought to evaluate the demographic patterns, mechanisms of injury, and clinical outcomes associated with CMF trauma in Puerto Rico and how cranial versus facial fractures influence injury severity and treatment. We hypothesize that in Puerto Rico, CMF trauma is more prevalent among adult men and is primarily caused by MVAs and falls; additionally, we believe patients with cranial and facial fractures will have significantly higher Injury Severity Scores (ISS). Epidemiological information gathered from this study will help direct public health efforts toward the prevention and enhancement of medical training for the adequate management of CMF traumas.

## Materials and methods

This study is a retrospective analysis of epidemiological and clinical data of 1,102 patients who were treated for CMF traumas at the Department of Trauma of the Administración de Servicios Médicos de Puerto Rico (ASEM) in the Medical Center of Puerto Rico from January 2018 to October 2022. The records of all patients (irrespective of sex, age, or outcome) who were treated for CMF trauma at ASEM during the study period were included based on the International Classification of Diseases, 10th Revision (ICD-10) diagnostic classification. Computed tomography (CT) imaging, including head, maxillofacial, and temporal bone protocols, was performed for the diagnosis of CMF fractures.

All the patients included in this study were from Puerto Rico. Patients were subdivided into age groups and sex to evaluate the injury prevalence and mechanisms. Patient data included personal information, mechanism of injury, type of fractures, type of cranial fractures, type of facial fractures, type of treatment, outcome, and morbidity scores. Age groups were divided based on decades, with the exception of those classified as pediatric (aged 0-14), adolescent (aged 15-18), and elderly (aged ≥70). Mechanisms of injury included pedestrian-related accidents, car-related MVAs, non-car-related MVAs such as motorcycles and all-terrain vehicles, gunshot wounds, assault, falls, and others (i.e., animal attacks). Fractures were divided into cranial or facial fractures. Cranial fractures were stratified into frontal bone fractures, parietal bone fractures, occipital bone fractures, and temporal bone fractures. Facial fractures were stratified into upper face (frontal bone) fractures, middle face (orbit, nose zygomatic complex, and maxilla) fractures, and lower face (mandible) fractures. Treatment was classified into surgical versus non-surgical management. The outcome was classified as alive at discharge or deceased during hospital admission. Finally, morbidity was assessed using the Glasgow Coma Scale (GCS) and the ISS.

The primary study variables (sociodemographic and topical interest) were analyzed statistically using frequencies and percentages for categorical characteristics. Continuous variables, on the other hand, were assessed using measures of central tendency (mean) and dispersion (±SD). Comparisons for the differences in the variables GCS, ISS, and age by the presence of facial and cranial fractures were assessed using the independent samples test. Results with p < 0.05 were considered statistically significant, and all analyses were conducted in IBM SPSS v.29 (IBM Corp., Armonk, NY, US). It must be noted that based on the retrospective nature of the study, data for all the variables assessed was not available for all patients. Therefore, the total number of subjects (n) may vary for each variable, and the analytical assessment for each variable was adjusted accordingly. The Institutional Review Board (IRB) of the University of Puerto Rico Medical Sciences Campus approved this protocol.

## Results

From January 2018 to October 2022, 1,102 patients were treated for fractures related to CMF traumas in Puerto Rico. The combined demographic characteristics are presented in Table 1. An evident difference in sex prevalence was noted, with male patients being the most affected compared to the female population, 83.1% to 16.4%, respectively. The mean patient age was 40.67 years, with ages spanning from 0 to 100 years.

**Table 1:** Sociodemographic and Epidemiological Characteristics of the Study Sample MVA: motor vehicle accident

Variable	n	%
Sex		
Male	916	83.1
Female	181	16.4
Unspecified	5	0.5
Age (years), mean (SD)	40.67 (19.23)	
Age (years)		
0-14	41	3.7
15-18	52	4.7
19-29	292	26.5
30-39	206	18.7
40-49	156	14.2
50-59	152	13.8
60-69	106	9.6
≥70	97	8.8
Cause of injury		
Pedestrian	203	18.4
Car-related accident	252	22.9
MVA (non-car)	260	23.6
Gunshot	115	10.4
Assault	59	5.4
Fall	176	15.9
Other/unspecified	37	3.4
Cranial fracture site	351	32.7
Frontal	112	10.6
Temporal	172	16.2
Parietal	83	7.8
Occipital	56	5.3
Facial fracture site	751	70.0
Lower face (mandible)	190	17.7
Middle face (maxilla, nose zygoma, and orbit)	665	61.8
Upper (frontal bone)	97	9.0
Surgical vs. conservative management		
Surgical management	193	19.8
Conservative management	721	74.17
Outcome		
Alive	972	88.2
Dead	130	11.8

As shown in Table 1, the largest age group affected was the young adult cohort, aged 19-29 (n = 292). The incidence of affected groups was followed in a decreasing fashion by the age cohorts as follows: 30-39 (n = 206), 40-49 (n = 156), 50-59 (n = 152), 60-69 (n = 106), and 70 or more (n = 97). Interestingly, the less impacted group was the pediatric population, with the youngest age group, 0-14 (n = 41), followed by the adolescent cohort aged 15-18 (n = 52).

**Figure 1:**
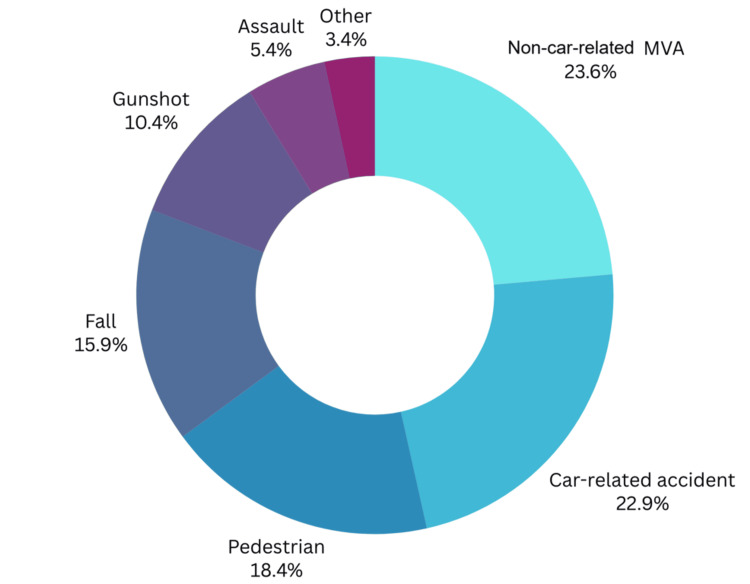
Mechanism of Injury Distribution MVA: motor vehicle accident

As depicted in Figure 1, the most common mechanism of injury was non-car-related MVA, such as motorcycle and four-track accidents (260, 23.6%), followed by car-related accidents (22.9%, 252), pedestrian-related accidents (203, 18.4%), and falls (176, 159%). The etiologies less responsible for CMF traumas were gunshot wounds (115, 10.4%); assault, such as fights and abuse (59, 5.4%); and other etiologies like animal attacks (37, 3.4%).

**Figure 2:**
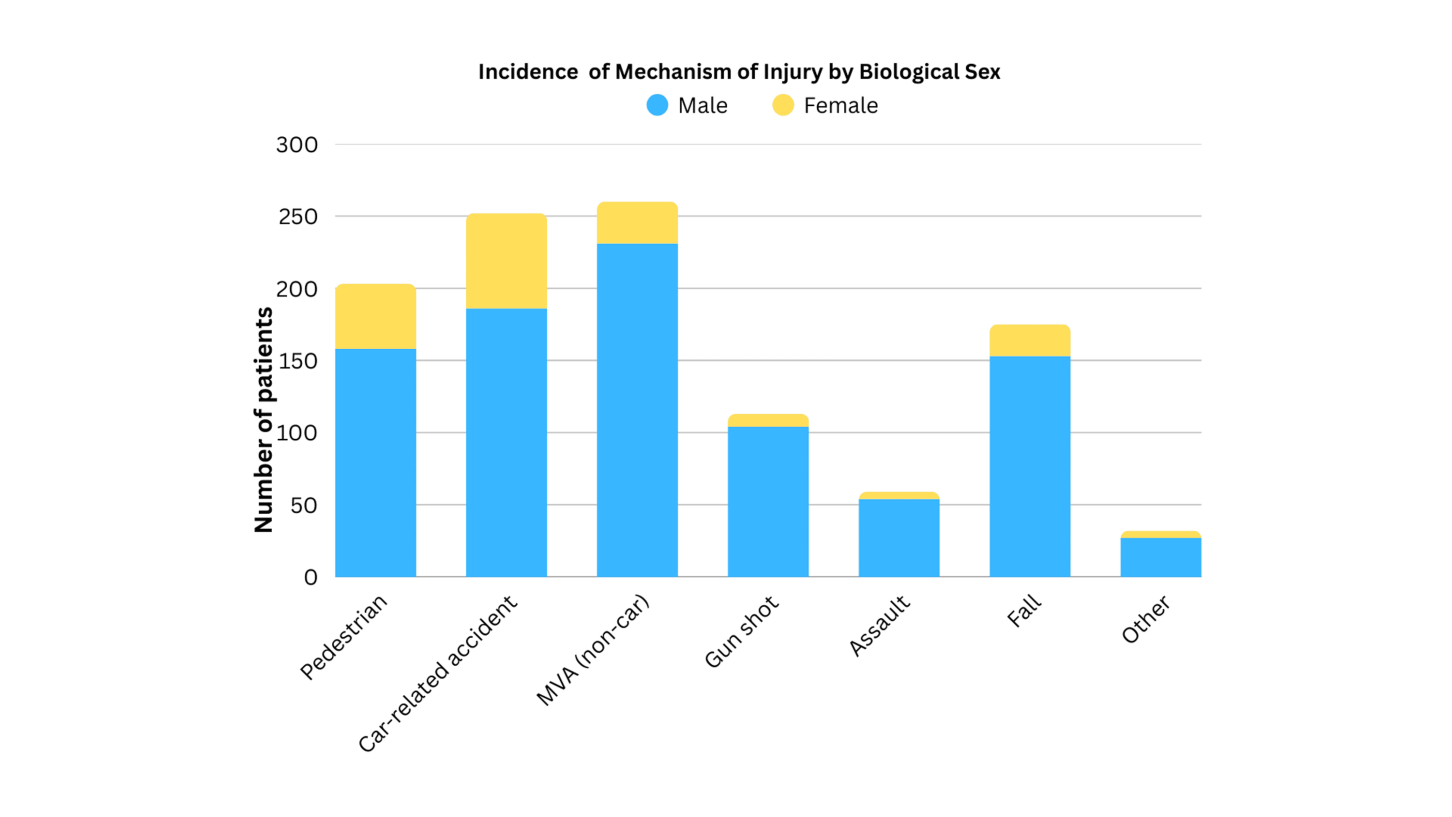
Comparison Between Mechanism of Injury and Sex MVA: motor vehicle accident

The primary mechanism of injury among the male population was non-car-related MVA (231, 25.3%) in contrast to the female population, in which car-related accidents were found to be the most common (66, 36.5%) (Figure 2). Compared to the male population, a considerable tendency toward pedestrian-related injuries was observed in the female group (17.3% vs. 24.9%, respectively). Falls occurred in a similar proportion between both groups, 16.8% (153) among the male group and 12.15% (22) among the female group. Increased risk among the male group toward gunshot (104, 11.4%) and assault-related injuries (54, 5.9%) was reported, while in the female group, both occurred in less proportion (5.0% and 2.8%, respectively).

**Figure 3:**
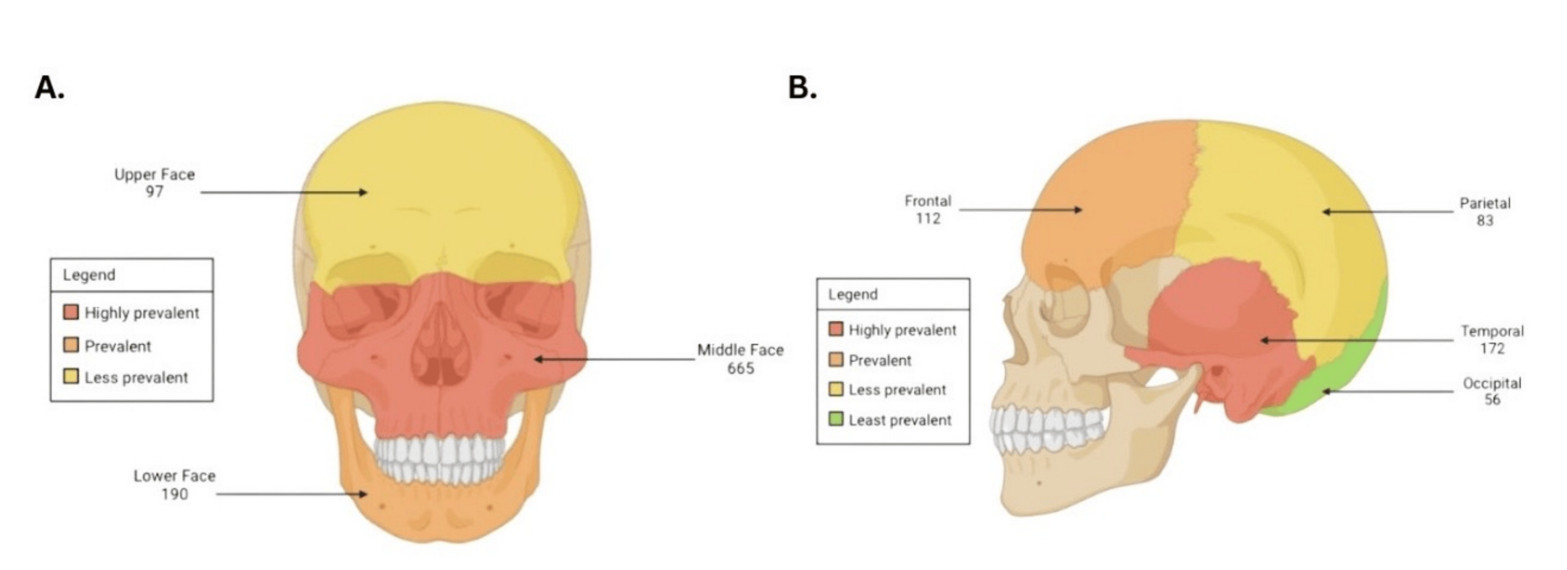
Anatomical Representation of the Incidence of Fractures per Facial and Cranial Regions This figure illustrates the incidence of fractures per facial and cranial region, categorized by specific anatomical zones. A color gradient was employed to depict the frequency of traumas at each site from the highest prevalent (red) to the least prevalent (green). (A) depicts facial traumas while (B) focuses on cranial traumas. The image was generated by the authors using BioRender.

A total of 751 (70.0%) patients presented with facial fractures, whereas cranial fractures were observed in 351 (32.7%) patients. When further detailing the type of facial fractures, the middle face was most frequently affected, accounting for 665 cases (61.8%). The lower face was affected in 190 cases (17.7%), whereas the upper face was involved in 97 cases (9%) (Figure 3A). When stratifying further by type of cranial fracture (Figure 3B), temporal fractures were the most frequently observed, found in 172 patients and accounting for 16.2% of cases. This was followed by frontal fractures at 10.6%, parietal fractures at 5.8%, and occipital fractures at 5.3%.

**Figure 4:**
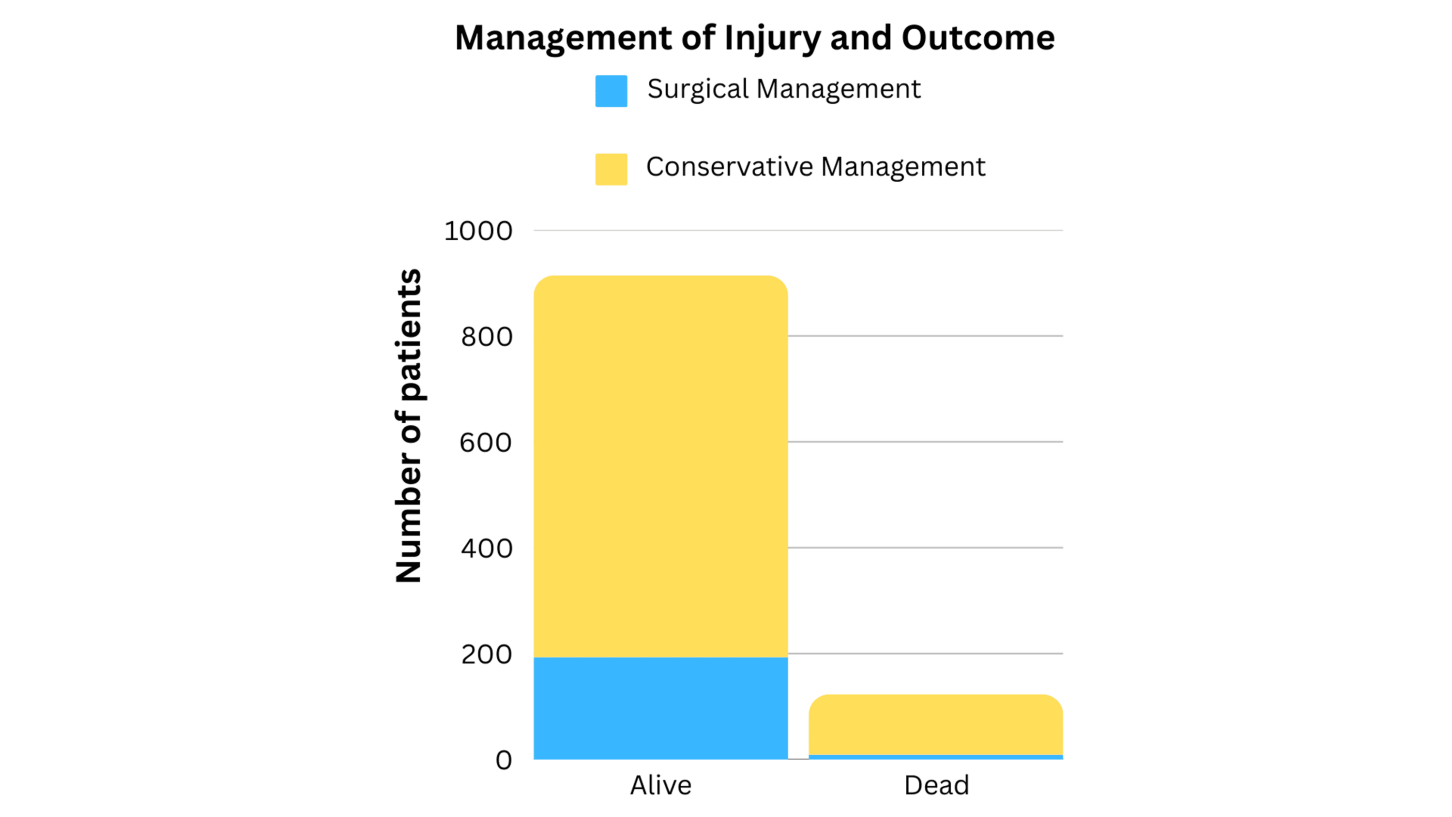
Comparison of the Type of Treatment and Outcome

When comparing the outcomes of all the patients involved in the study, 972 (88.2%) patients were alive at the time of discharge, while 130 (11.8%) cases passed away during hospital admission (Figure 4). Among the patients who remained alive during admission, 193 (19.8%) underwent surgery, and 721 (74.17%) had non-operative management. In cases where patients passed away during their hospital stay, nine (4.66%) underwent surgery, while 114 (87.7%) were managed conservatively.

**Figure 5:**
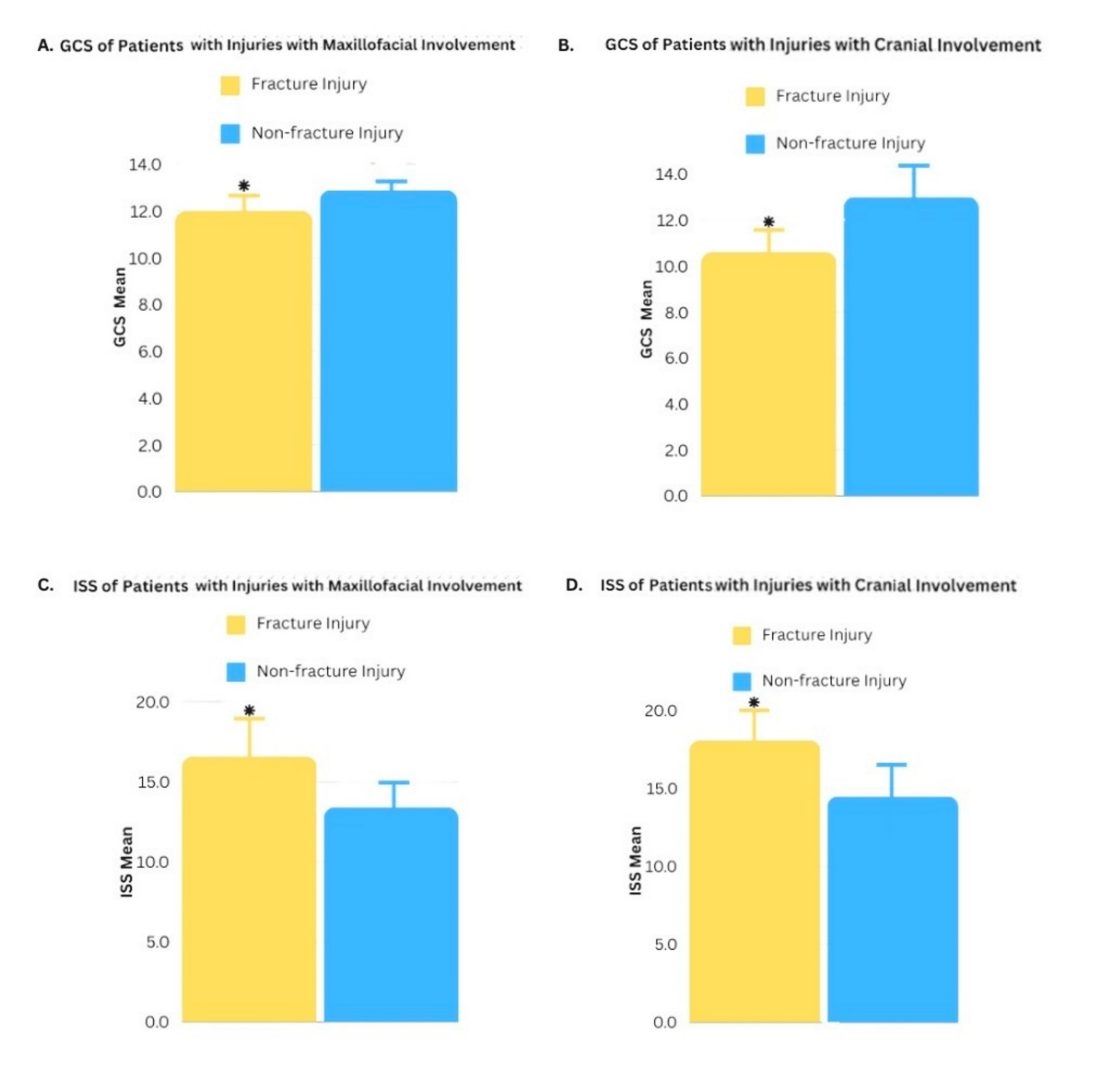
Glasgow Coma Scale (GCS) and Injury Severity Score (ISS) of Patients with Maxillofacial and Cranial Fractures This figure set compares the GCS and ISS of patients with maxillofacial or cranial fractures against those who did not suffer from said fractures in the same study sample. (A) demonstrates the GCS of patients with versus without maxillofacial fractures. (B) depicts the GCS of patients with versus without cranial fractures. (C) compares patients with maxillofacial fractures similarly yet in regard to ISS. Lastly, (D) compares the ISS of patients with versus without cranial fractures.

Patients with cranial fractures had a statistically lower (p < 0.001) average GCS of 10.6 compared to patients without cranial fractures (13.0) (Figure 5A). Likewise, patients with facial fractures had a statistically lower (p < 0.001) average GCS of 12.0 compared to patients without facial fractures (12.9) (Figure 5B). Patients with cranial fractures had a statistically higher (p < 0.001) ISS, with an average score of 18.14, in comparison to 14.6 among patients without cranial fractures (Figure 5C). The average score of ISS with facial fractures (16.6) was also found to be statistically higher (p < 0.001) when compared with patients without facial fractures (13.5) (Figure 5D).

## Discussion

This study was motivated by the significant gap in data on the prevalence of CMF traumas, leading to our aim of understanding the prevalence, sociodemographic, and clinical characteristics of CMF traumas in Puerto Rico. This research is pivotal for identifying at-risk populations and common fracture patterns while optimizing treatment approaches for CMF traumas within our community.

Our research indicates that CMF injuries commonly occur in young male patients, particularly those in their 20s and 30s (Table 1). Our results also showed a higher predominance of CMF injuries in the male group with a male-to-female ratio of 5:1 for CMF injuries, consistent with worldwide numbers that range from 2:1 to 32:1 [7-13]. These gender-based differences were further underscored in Figure 2, showcasing that the male population was predominantly affected in every category. The male group was more likely to be involved in non-car MVAs and the female group in car-related accidents. This could reflect trends in behavioral patterns and occupational exposures. For example, men might be more inclined to ride motorcycles or four-tracks or engage in riskier driving when women are passengers. In terms of falls, a similar proportion was seen. However, there was an increased risk in incidence observed in the gunshot, assault-related injury, and other/unspecified categories. The increased risk among the male population for gunshot and assault-related injuries draws attention to the role of social determinants of health and possible inclination to violent behavior.

Study findings also highlight that the frequency of CMF fractures is highest among individuals between 19 and 29 years old, with a gradual decline in prevalence observed as the age of the patients increases. Consistent with our results, multiple studies have reported the highest prevalence of CMF injuries in patients younger than 35, particularly those in their 20s (i.e., 20-29) [12]. The predominance of young male patients with CMF injuries most likely stems from partaking in high-risk behaviors (i.e., reckless driving and not wearing protective gear) and inclination toward outdoor activities [12].

As for the etiology of these injuries, our study revealed that non-car-related MVAs, such as motorcycles and four-track vehicles, were the primary causative agents of CMF fractures, accounting for 23.6% of cases (Figure 1). Car-related accidents closely followed this at 22.9% and pedestrian incidents at 18.4%. Collectively, motor vehicles were associated with 46.5% of CMF fractures in this population, highlighting their significant role in these injuries. These findings underscore the importance of MVAs as a primary cause of CMF trauma. However, regional variations in the causes of CMF injuries have been observed in previous studies.

A retrospective study conducted at a level 1 trauma center in Atlanta (2015-2018) identified assault as the leading cause of CMF fractures among over 1,000 patients (44%), exceeding motor-related accidents (22%) and motorcycle accidents (0.8%) [10]. This disparity could be partly due to robust seatbelt enforcement, advanced airbag systems, and vehicle safety technologies in the US mainland, which have successfully reduced the severity of motor vehicle-related injuries. While these measures have reshaped the trauma landscape by decreasing motor-related CMF injuries, they have little influence on assault-related cases, which are more affected by social factors like urban crime and community violence. Another possible explanation may be road and structural conditions, which may be less well-developed in Puerto Rico. Alternatively, a study in northern Israel found falls to be the leading cause of CMF fractures (45.4%), with MVAs close second at 39.2% [11]. However, aligning more closely with our results, an epidemiological study at Tong University School of Medicine in Shanghai, China, indicated that traffic accidents were the primary cause of injury, responsible for 48.95% of CMF fractures, followed by falls at 21.10% [14]. It is crucial to acknowledge that regional differences in CMF injury causes may also stem from various factors, such as differing traffic laws, the prevalence of specific vehicle types, and cultural attitudes toward road safety. For instance, in some European countries with higher cycling rates, bicycle-related CMF injuries may be more common than in areas with less developed cycling infrastructure. Meanwhile, transportation in Puerto Rico is heavily dependent on automobiles.

When determining the clinical characteristics of CMF traumas in Puerto Rico, we first divided the study results into two distinct groups: maxillofacial versus cranial trauma. As noted in Table 1, facial traumas were much more common than cranial traumas in our patient population. These findings are consistent with a study performed by Siswanto and Hua in 2012, where they sought to analyze the strength of the human skull in high-speed impact injuries [15]. Although this study attributed the greatest strength score to the side mandible, this was closely followed by the temporal and frontal bones, then the maxilla, and afterward the occipital and parietal bones, with the rest of the bones assessed being weaker than these structures. Overall, the cranial bones as a group had greater strength scores than facial bones, mimicking the findings included in our study.

Upon assessment of the maxillofacial structures affected by trauma, the most commonly affected site was the middle face as portrayed in Figure 3A. These findings are in agreement with those from a study performed by Hwang and You in 2010, where they analyzed facial bone fractures in a large patient population [16]. This study found that the most common isolated facial fracture site was the nasal bone, followed by the mandible, orbital bones, zygoma, maxilla, and lastly, the frontal bone [16]. These findings are consistent with those in our study, where the most commonly affected facial bones were from the middle face, followed by the lower face, and then the upper face. This may be partially due to the fact that the nose is the most prominent facial feature, which is highly exposed in traumatic injuries, leading to a higher prevalence of middle-face injuries. However, it must be noted that our study did not stratify facial fracture sites by specific bone fractures, which may provide more detailed information regarding the most commonly affected structures in these areas. Nonetheless, as a whole, the findings are consistent with those reported in the literature. Additionally, the study by Siswanto and Hua found that the greatest strength score for facial structures was attributed to the side mandible, with the weakest scores being the front mandible and the nasal bone, which once again coincides with the results from our study in terms of cataloging the middle face structures as those most prone to injury [15].

Regarding cranial bone fractures, temporal bone injuries were the most common, followed by frontal bone fractures, as demonstrated in Figure 3B. According to the previously mentioned study, the temporal and frontal bones are considered to be the strongest of the cranial bones, while the parietal bone is considered to be the weakest [15]. Although this challenges the results of our study in which parietal bone fractures were the third most common, these results may be partially explained by the etiology of trauma suffered by the patient population instead of the relative strength of the cranial bones individually. A study performed by Amin et al. in 2008 demonstrated that fractures of the temporal and frontal bones were associated with severe MVAs [17]. These results go in accordance with the findings in our study, since the most common cause of trauma in our cohort was MVAs. Therefore, the higher incidence of MVAs in our patient cohort accounts for the higher percentage of temporal and frontal bone fractures seen in our study.

Beyond skeletal injury, CMF trauma may also compromise vascular structures. Recent literature has revealed that blunt cephalovascular trauma may be present in these patients, which is rare and often missed due to subtle symptoms unless transection of vascular structures is involved [18]. This study found that the superficial temporal artery was most commonly injured, with pseudoaneurysms being the most common injury. These vascular injuries were mainly treated with surgical ligation or endovascular embolization. They concluded that a high suspicion for blunt cephalovascular injury warrants a CT angiography (CTA), stressing the importance of employing standardized protocols to help identify and manage vascular injuries in CMF trauma patients [18].

Our study findings demonstrate that most patients who survived their hospital stay underwent non-operative management rather than surgical interventions (Figure 4). This finding is consistent with existing literature indicating that most craniofacial fractures encountered at level 1 trauma centers do not necessitate surgical intervention, as highlighted by Spinella et al. [19]. This alignment underscores the importance of recognizing that most craniofacial fractures can be effectively managed without surgery. Acknowledging this fact when planning and delivering treatment to patients is essential. This can reduce the burden on patients and the healthcare system while ensuring optimal outcomes and personalized care.

In CMF trauma management, the decision regarding whether surgical intervention is warranted remains a complex and multifaceted issue. Different from some medical conditions where treatment protocols are well-established, there exists no consensus in the literature on when surgery should be pursued for facial fractures. This lack of consensus stems from the inherent variability and complexity of each case, including factors such as fracture type, patient comorbidities, previous medical conditions, and the mechanism of injury. However, the Spinella et al. study observed that mandible fractures were more likely to require surgery, mainly when occurring in isolation or in combination with other fractures [19]. Additionally, they found that patients under the age of 50 exhibited a higher propensity for surgical intervention compared to older individuals. These findings highlight the critical importance of conducting a thorough and individualized assessment for each patient, considering factors such as fracture location and patient age. Such an approach is crucial for determining the need for surgical intervention in craniofacial fracture cases and ensuring optimal treatment outcomes.

In order to assess morbidity in our study, the GCS and ISS were used. In terms of the former, this widely used scale objectively describes the level of consciousness of patients after trauma and acute medical events. The three parameters used in the scale are eye-opening, verbal, and motor response. Thus, this scoring system adequately summarizes the patients' current clinical picture [20]. The utility of the GCS relies on the ability to guide emergent management in trauma or acutely sick patients and to serve as a tool to follow the clinical response of patients [21]. For example, in terms of maxillofacial fractures, our data reflects with statistical significance that patients with these injuries also had lower GCS values when compared to patients without facial fractures (Figure 5A). Furthermore, Figure 5B shows that patients who had cranial fractures presented with significantly lower GCS scores when compared to patients who did not have cranial fractures. Lower GCS values (<6.5) correlate with higher mortality in patients [22]. In our study, the mean was above the indicated value, thus correlating to the low mortality rate in our patient cohort. Therefore, although the CMF fractures in our study group ranged from mild to severe, most of the patients had a high GCS value, low mortality, and high survivability.

On another note, the ISS is an anatomical scoring system that provides an overall score for patients with multiple injuries and correlates linearly with mortality, morbidity, and other measures of severity [20]. The ISS is a sum of the three largest abbreviated injury scale (AIS) scores. ISS ranges from 0 to 75, with values equal to or larger than 15 representing “major trauma.” ISS reflects a direct proportional relationship when compared to GCS. Using the ISS score to correlate mortality is challenging because it considers other factors, such as age and comorbidities [23]. However, it is helpful for a holistic understanding of the injuries when taken into account together with other variables, such as GCS, clinical factors, and statistical data. Our study shows that patients with both maxillofacial (Figure 5A) and cranial (Figure 5B) fractures had higher ISS values when compared to patients without fractures, with many having traumas categorized as “major traumas.” Thus, it can be said that patients with cranial and/or facial fractures had more severe injuries and worse clinical status than those who suffered from CMF traumas without fractures. Similarly, a study by Lupascu et al. found that facial fractures were significantly associated with higher rates of traumatic brain injury compared to patients without facial fractures [24]. It must be noted that many patients included in our cohort may have also suffered from polytrauma, including traumatic brain injury, which may also affect patient outcomes, including mortality rates.

Although our study serves as the first source of information regarding CMF traumas in the Puerto Rican population, there are several limitations associated with our study design. First, this is a single-center study of a small duration; therefore, even though there is an adequate sample size, said findings may not be representative of the entire population since factors such as accessibility and geographic location may influence the patient population served at this institution. A longer duration of the study period would also allow for a bigger patient pool, facilitating stronger conclusions regarding the etiologies of CMF traumas and aiding in the development of management strategies that take these factors into account. Additionally, there is only one trauma center in Puerto Rico, for which management and outcomes are solely dependent on one institution, which may lead to biases. On another note, the institution in question transitioned to an electronic medical record in the past few years, for which there is missing data regarding CMF traumas in the Puerto Rican population due to the recent integration of online medical records and poor familiarity with the use of said system. Along said lines, data such as the presence of comorbidities may be lacking due to the absence of standardized protocols for patient charting, which complicates the data extraction process. Further evaluation of patient records is required to account for said variables and determine if the presence of comorbidities may affect patient outcomes. In addition, many trauma patients at the study institution suffer from polytrauma, including traumatic brain injury and blunt cephalovascular injuries, which may affect patient outcomes as previously detailed, including mortality rates, and this was not taken into account in our analysis. It must be noted that our study does not consider the variability of mechanisms of injury specific to each age cohort. Instead, we focused on highlighting the overall patterns of injury for the patient cohort as a whole. Therefore, trends in terms of mechanisms of injury may vary with patient age, which is not being addressed by our study. Lastly, although our study did evaluate CMF traumas based on their overall location, we did not subdivide each classification and assess bones individually, which could provide additional information as to the most commonly fractured bones instead of their relative location and interventions associated with each fracture location in particular.

## Conclusions

To our knowledge, this is the first study to identify and characterize CMF fractures in the Puerto Rican population. Similar to other studies, most patients suffering from CMF fractures were adult men, whereas the etiologies of injury were consistent with those previously reported in the literature. The temporal bone and middle face were the areas most affected in the skull and face, respectively. On another note, facial fractures were more prevalent than cranial fractures. It must be noted that most patients were managed non-surgically, which is a common trend in the literature. This data serves as a stepping stone to guide public health funding toward the prevention and treatment of CMF traumas in the Puerto Rican population.
